# Subtle white matter alterations in schizophrenia identified with a new measure of fiber density

**DOI:** 10.1038/s41598-019-40070-2

**Published:** 2019-03-15

**Authors:** Philipp Stämpfli, Stefan Sommer, Andrei Manoliu, Achim Burrer, André Schmidt, Marcus Herdener, Erich Seifritz, Stefan Kaiser, Matthias Kirschner

**Affiliations:** 1 Department of Psychiatry, Psychotherapy and Psychosomatics, Psychiatric Hospital, University of Zurich, Zurich, Switzerland; 2 MR-Center of the Psychiatric Hospital and the Department of Child and Adolescent Psychiatry, University of Zurich, Zurich, Switzerland; 3 University of Basel, Department of Psychiatry (UPK), Basel, Switzerland; 4 Center for Addictive Disorders, Department of Psychiatry, Psychotherapy and Psychosomatics, Psychiatric Hospital, University of Zurich, Zurich, Switzerland; 5 Division of Adult Psychiatry, Department of Mental Health and Psychiatry, Geneva University Hospitals, Geneva, Switzerland; 6 Montreal Neurological Institute, McGill University, Montreal, Quebec Canada; 7 Wellcome Centre for Human Neuroimaging, University College London, London, United Kingdom; 8 Max Planck University College London Centre for Computational Psychiatry and Ageing Research, London, United Kingdom

## Abstract

Altered cerebral connectivity is one of the core pathophysiological mechanism underlying the development and progression of information-processing deficits in schizophrenia. To date, most diffusion tensor imaging (DTI) studies used fractional anisotropy (FA) to investigate disrupted white matter connections. However, a quantitative interpretation of FA changes is often impeded by the inherent limitations of the underlying tensor model. A more fine-grained measure of white matter alterations could be achieved by measuring fiber density (FD) - a novel non-tensor-derived diffusion marker. This study investigates, for the first time, FD alterations in schizophrenia patients. FD and FA maps were derived from diffusion data of 25 healthy controls (HC) and 21 patients with schizophrenia (SZ). Using tract-based spatial statistics (TBSS), group differences in FD and FA were investigated across the entire white matter. Furthermore, we performed a region of interest (ROI) analysis of frontal fasciculi to detect potential correlations between FD and positive symptoms. As a result, whole brain TBSS analysis revealed reduced FD in SZ patients compared to HC in several white matter tracts including the left and right thalamic radiation (TR), superior longitudinal fasciculus (SLF), corpus callosum (CC), and corticospinal tract (CST). In contrast, there were no significant FA differences between groups. Further, FD values in the TR were negatively correlated with the severity of positive symptoms and medication dose in SZ patients. In summary, a novel diffusion-weighted data analysis approach enabled us to identify widespread FD changes in SZ patients with most prominent white matter alterations in the frontal and subcortical regions. Our findings suggest that the new FD measure may be more sensitive to subtle changes in the white matter microstructure compared to FA, particularly in the given population. Therefore, investigating FD may be a promising approach to detect subtle changes in the white matter microstructure of altered connectivity in schizophrenia.

## Introduction

In the last decades, advanced magnetic resonance imaging (MRI) techniques have started to shed light on the complex brain abnormalities observed across all stages of schizophrenia^1–3^. Convergent findings from functional and structural imaging studies suggest that alterations in cerebral connectivity may be the core pathophysiological mechanism underlying the development and progression of information processing deficits in schizophrenia^4–7^. In recent years, diffusion-weighted imaging methods were used to investigate the white matter (WM) integrity as anatomical correlate of this disconnection hypothesis^1,8^. The most commonly assessed parameter in DTI studies is the fractional anisotropy (FA). Emerging evidence suggest that connectivity in frontal regions seems to be most affected^1,9–12^. Critically, FA alterations in these frontal tracts, including the thalamic radiation (TR), the superior longitudinal fasciculus (SLF), and arcuate fasciculus, have been related to positive psychotic symptoms and deepens our understanding of the relation between disconnectivity and symptom expression^13–19^.

However, it has been shown that the tensor model and therefore FA measures are inadequate to characterize the underlying tissue structure in regions with complex fiber geometries and multiple fiber populations^20–25^. Voxels capturing these anatomical complexities occur frequently (60%-90% of all WM fiber voxels) throughout the brain WM due to limited spatial resolution and partial volume effects between adjacent tracts^25^. Although a reduction of FA is often thought to reflect axonal degeneration and demyelination^26–29^, complex fiber configurations may critically confound the interpretation of changes in tensor-derived diffusion metrics and complicate a correct and quantitative interpretation of changes in DTI-related parameters^25,30–34^.

In the last few years, diffusion acquisition techniques and data analysis pipelines improved significantly by addressing these inherent problems of the tensor model. Higher order diffusion models based on acquisition schemes with high number of diffusion directions and new reconstruction methods as the constrained spherical deconvolution technique^35^ were developed. These advances enable the resolution multiple fiber directions within a single voxel and improved the performance of fiber tractography significantly^36–38^. Additionally, there is a growing literature on removing tractography biases which is a crucial prerequisite to derive absolute and quantitative measures from fiber tractograms^39,40^.

However, the reliable extraction of quantitative measures from tractograms across different populations remains challenging. Recent developments in global top-down tractography optimizations enable the estimation of fiber contributions and compartment fractions^41–46^, whereby all of these optimization methods have their own pitfalls (please see the article of Daducci and colleagues for a comprehensive review^47^). Numerous models based on diffusion weighted imaging have been proposed to estimate parameters related to the restricted, intra-axonal compartment, commonly referred to as fiber density (FD)^48,49^. In the work of Smith *et al*. and Daducci and colleagues^44,46^, an optimal weight for each streamline is determined according to a biologically motivated forward model and the measured diffusion signal. By assigning a weight of zero, false positive or implausible connections can be eliminated. The FD is calculated by multiplying each streamline contribution (fiber weight) by the streamline length.

Previously, we have demonstrated that white matter alterations were more prominent in FD compared to FA in patients with a progressive neurological disease (amyotrophic lateral sclerosis)^50^.

However, the potential advantages of the aforementioned innovations in basic neuroimaging methods have not been established for clinical neuroscience research of pathologies in schizophrenia. It is unknown whether microstructural white matter fiber changes can be quantified with the new FD measure. Therefore, in the present study, we applied a novel whole brain TBSS analysis approach^51^ to test its potential in detecting microstructural alterations in FD between patients with schizophrenia and healthy controls. We hypothesize that, compared to FA, the new FD measure will be more able to detect microstructural WM changes in schizophrenia. Furthermore, based on previous findings^13,14^, we investigated the relation between FD changes in the frontal fasciculi and positive psychotic symptoms to explore the association between microstructural changes and symptom expression.

## Materials and Methods

### Participants

We recruited patients with chronic schizophrenia (SZ) (n = 21) from inpatient and outpatient units of the Psychiatric University Hospital in Zurich and from affiliated institutions. Healthy controls (HC) (n = 25) were recruited from the general community. Inclusion criterion for patients with SZ was schizophrenia or schizoaffective disorder confirmed with the structured Mini International Neuropsychiatric Interview for DSM IV (MINI). Individuals with any other Axis I DSM IV disorder, in particular major depression or current substance use disorder, were excluded from the study. All individuals with SZ were clinically stable and received a stable dose of second-generation antipsychotic, with no change in medication dose for at least two weeks. The study was approved by the local ethics committee of the Canton Zurich. All participants signed the written informed consent in accordance with the Declaration of Helsinki. The capability of all patients to give informed consent was evaluated by the treating psychiatrist. One patient with SZ had to be secondarily excluded due to insufficient data quality caused by excessive head motion (see section on data quality assessment). Thus, 20 chronic SZ patients were included in the subsequent analysis.

### Psychopathological and neuropsychological assessment

All study participants underwent an extensive psychopathological and neuropsychological assessment. We assessed symptom severity with the Positive and Negative Syndrome Scale (PANSS)^52^ and functioning with the Global Assessment of Functioning scale (GAF)^53^. Moreover, all participants performed a comprehensive neuropsychological test battery, which has been used in previous studies^54–57^. We assessed verbal learning (Auditory Verbal Learning Memory Test)^58^ verbal and visual short-term working memory^59^, Corsi block-tapping^60^, processing speed (Digit-Symbol Coding)^61^, planning (Tower of London)^62^ and semantic and phonetic fluency (animal naming, s-words)^63^. Results of all cognitive tests were summarized in a composite cognition score computed with the mean of z-transformed scores (based on HC group data). Additionally, we used the Multiple Word Test^64^ to control for premorbid verbal intelligence.

### MRI data acquisition

MRI data acquisition was performed on a 3T whole-body MR scanner (Achieva, Philips Healthcare, Best, the Netherlands), equipped with 80 mT/m gradients and a 32-channel receive head coil. Diffusion data were acquired using a diffusion-weighted single-shot spin-echo echo-planar imaging sequence (ssh SE-EPI sequence) with the following parameters: repetition time (TR) = 6.64 s, echo time (TE) = 53.6 ms, field of view (FOV) = 240 × 240 mm^2^, 50 contiguous transversal slices, slice thickness = 2.5 mm, acquisition matrix = 96 × 96, SENSE factor = 2.5, partial Fourier encoding = 60%. The slices were positioned parallel to the anterior and posterior commissure defined on a T_1_-weighted midline sagittal survey image. Diffusion acquisition was performed along 32 directions with a b-value of 1000 s/mm^2^ and two signal averages (NSA = 2). Additionally, 4 non-diffusion-weighted b = 0 s/mm^2^ scans were acquired resulting in a scan time of 8 min 31 s. For structural reference and anatomical priors for the tracking algorithm, T_1_-weighted images were recorded using a three-dimensional magnetization prepared rapid gradient-echo (MP-RAGE) sequence with 1 mm isotropic resolution.

### Data quality assessment

**Figure 1:**
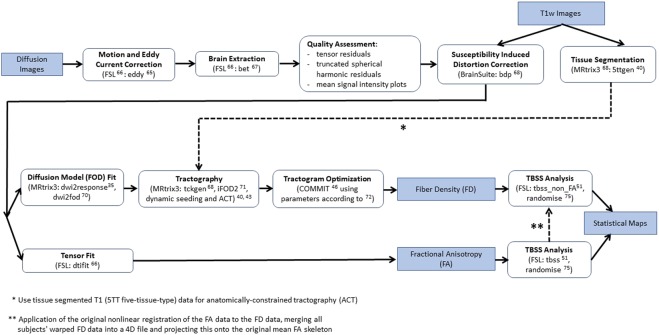
Data processing pipeline. All data processing steps performed on the diffusion and the T1 weighted anatomical data are illustrated in the flowchart. All tools and parameters applied on the raw data up to the final statistical maps are described and referenced in detail.

All data processing steps are illustrated in Fig. 1. First, diffusion data was corrected for “eddy” current-induced image distortions and subject motion using the eddy tool^65^ in FSL (Analysis Group, FMRIB, Oxford, UK, version 5.0.6)^66^. The brain extraction tool from FSL^67^ was applied to remove non-brain tissue from the diffusion data and to estimate the inner and outer skull surfaces. Then, quality control of all diffusion data was assessed based on several criteria: First diffusion tensor residuals were calculated for every acquired diffusion direction and the nine slices in the whole diffusion dataset with the highest residuals were identified for visual inspection. Second, the MRtrix3 software package (http://www.mrtrix.org)^68^ was used to estimate the voxel-wise noise using the residuals from a truncated spherical harmonics fit. Plots were generated depicting the twelve slices with the highest noise level, four in sagittal, four in axial, and four in coronal direction, respectively. Third, mean signal intensity plots for every diffusion direction and the non-diffusion-weighted image were derived and plotted slice by slice in sagittal, axial, and coronal directions. Artifacts such as signal dropouts due to head motion can easily be spotted on these plots. Two trained MR physicists separately inspected the data for artefacts and rated the signal courses and fitting residuals of every subject on a Likert-type scale. Only one patient showed severe signal dropouts in multiple diffusion directions and several slices caused by head motion. Consequently, this patient was an outlier in the overall sum of the Likert-scale quality ranking scores and had to be excluded for the subsequent analyses.

### Diffusion data analysis and parameter calculation

#### FD and FA Computation

In order to improve alignment to the T_1_-derived tissue priors, the diffusion data were corrected for susceptibility-induced image distortions^69^. Afterwards, constrained spherical deconvolution with recursive calibration of the response function^35,70^ and fiber tractography was performed in MRtrix3^68^ using the iFOD2 probabilistic tractography algorithm^71^. In order to apply biological tissue priors to the streamline generation, the “Anatomically-Constrained Tractography” (ACT) option was selected (MRtrix3 tckgen act option)^40^. Tractography seed points were determined dynamically according to the spherical-deconvolution informed filtering of the tractogram model (MRtrix3 tckgen seed-dynamic option for determining seed points based on the SIFT model^43^). Due to this dynamic seeding strategy within the whole white matter, the distribution of streamlines is already approximating the apparent fiber density and therefore, intrinsic tractography biases are reduced. In total, five million fibers were generated per subject.

The resulting streamlines were optimized using the COMMIT framework^46^ applying the parameters described in the article from Sommer and colleagues^72^. The derived intracellular compartment fraction corresponds to the FD. Furthermore, FA maps of every dataset were calculated with the FSL software package^66^.

### Comparison between intra-subject variability of FD and FA

In order to provide evidence that the intra-subject variability and reproducibility of the FD measure is comparable to the FA^73,74^, we assessed the coefficients of variation (CoV) of both measures in an independent longitudinal data set of 36 healthy subjects. Each subject was measured at two time points with 7 days between the first and second scan. Details of this analysis can be found in the supplementary materials section. In brief, the CoV of the FD and FA were small with mean values less or equal 2% (Table S1). As described previously for the FA by Vollmar *et al*. and Veenith and colleagues^73,74^, these findings provide first evidence for high intra-subject reproducibility of the new FD measure in longitudinal data.

### TBSS analysis

TBSS analysis was performed to assess differences between the healthy control group and patients with chronic SZ using the standard processing steps as described by Smith and colleagues^51^. First, all native FA maps were nonlinearly registered to a 1 × 1 × 1 mm^3^ MNI152 template. The FA maps were averaged to create a mean FA map, which was thereafter skeletonized to build a FA skeleton that represents the main tracts common to all subjects. Finally, this FA skeleton was thresholded at a FA value of 0.2.

The FD images were analyzed using the “tbss_non_FA” command (as described in the TBSS user guide (https://fsl.fmrib.ox.ac.uk/fsl/fslwiki/TBSS/UserGuide)). This step is the standard algorithm of the TBSS framework for scalar diffusion maps other than FA maps. In other words, the FD images were analyzed with the same nonlinear registration-, warping-, and skeleton projection operations used during the processing of the corresponding FA images^51^.

To evaluate differences between the groups, voxel-wise analysis based on a general linear model was performed using FSL’s randomize tool^75^ with 5000 permutations to correct for multiple comparisons (age and gender were used as regressors of no interest in the design). A family wise error (FWE) corrected p-value of p < 0.05 was considered statistically significant. The TBSS results included threshold-free cluster enhancement^76^. Two contrasts were computed, testing for positive and negative differences of the FA and FD parameters between the healthy control and the patient group. Furthermore, we addressed potential associations between fiber density and current medication dose in all clusters showing significant FD differences in the TBSS analysis between HC and SZ by correlating the FD values with chlorpromazine equivalents (mg/d).

### Correlation Analysis with positive psychotic symptoms

In addition to the whole brain TBSS analysis, we performed a region of interest (ROI) analysis to evaluate correlations between alterations in fiber density and symptom severity in patients with chronic SZ. The ROI analysis was based on previous DTI studies investigating the association between WM changes and positive psychotic symptoms in frontal fasciculi^13,14^. Within these frontal regions, we restricted our ROI analysis to regions showing FD differences between HC and patients with chronic SZ in the whole brain analysis. The following five fasciculi were included: the left and right thalamic radiation (TR), the left and right superior longitudinal fasciculus (SLF), and the right uncinate fasciculus (UF). As opposed to the TBSS method, the ROI approach estimates the integrity of entire ROIs, rather than the central integrity of the tracts only and has the advantage of a higher regional sensitivity as compared to a whole brain, voxel-based method.

#### ROI definition

The five anatomical ROIs defining these five major fiber tracts were obtained from a probabilistic tractography atlas (JHU white-matter tractography atlas^77^). In total, the JHU white-matter tractography atlas describes 20 major fiber bundles. The probability-weighted mean FD values were calculated for each of the five fiber tracts by multiplying the probability-weighted maps of the individual tract with co-registered FD maps from the TBSS analysis. In other words, the probability tract maps served as ROIs to extract the FD values within each of the five fiber bundles. These mean FD values were then correlated with the PANSS positive score of patients with chronic SZ. Bonferroni correction was applied to correct for multiple comparison across the five predefined anatomical ROIs.

## Results

### Demographics and clinical Data

For demographic and clinical data please see Table 1. Patients with chronic SZ and HC did not differ in age, gender, handedness, educational years and premorbid verbal intelligence (MWT IQ). In contrast, we found a significant difference in the composite cognition score derived from our neuropsychological test battery.

**Table 1:** Demographic, Psychopathological and Clinical Data.

	Healthy Controls (n = 25)	SZ patients (n = 20)	Test statistics	*p* value
Age	32.1 (8.6)	32.7 (8.3)	*U* = 2*36*.*0*	0.749
Gender (f, m)	9, 16	4, 16	*Χ*^2^ = *1*.*385*	0.239
Handedness (r, l)	21, 4	16, 4	*χ*^2^ = *0*.*122*	0.725
Education, Years (SD)	12.5 (3.5)	12.1 (3.5)	*U* = *239*.*5*	0.806
Duration of illness, Months (SD)^a^		119 (87.8)		
Chlorpromazine Equivalents (mg/d)		493.3 (379.9)		
**Psychopathology**				
PANSS Total		48.9 (11.3)		
PANSS Positive		10.7 (2.4)		
PANSS Negative		15.3 (5.9)		
PANSS General		22.9 (5.3)		
BNSS Total		25.7 (12.2)		
BNSS Apathy^b^		16.1 (6.5)		
BNSS Diminished Expression^c^		9.6 (7.5)		
GAF		55.3 (11.1)		
**Cognition**				
Cognition Score^d^	0.06 (0.53)	−0.44 (0.94)	*t* = 2.025	0.054
MWT IQ	27.8 (4.1)	26.7 (6.2)	*t* = 0.657	0.516

*Note*: Data are presented as means and standard deviations. Group differences were investigated using 2-sample t tests for continuous and *χ*^*2*^ tests for categorical data. For non-normally distributed data Mann–Whitney *U* tests were applied. PANSS, Positive and Negative Syndrome Scale; BNSS, Brief Negative Symptom Scale; GAF, Global Assessment of Functioning; MWT IQ, Multiple Word Test Intelligence Quotient. ^a^Duration of illness included the duration of untreated psychosis and the time period since initiation of treatment. ^b^BNSS Apathy = Avolition, Anhedonia, Asociality; ^c^BNSS Diminished Expression = Affective Flattening or Blunting, Alogia. ^d^Cognition data were z-transformed based on the data of the HC group for each test separately. The Composite cognition score was computed as the mean of the z-transformed test scores on subject level.

### Differences in FD and FA value between HC and patients with chronic schizophrenia

**Figure 2:**
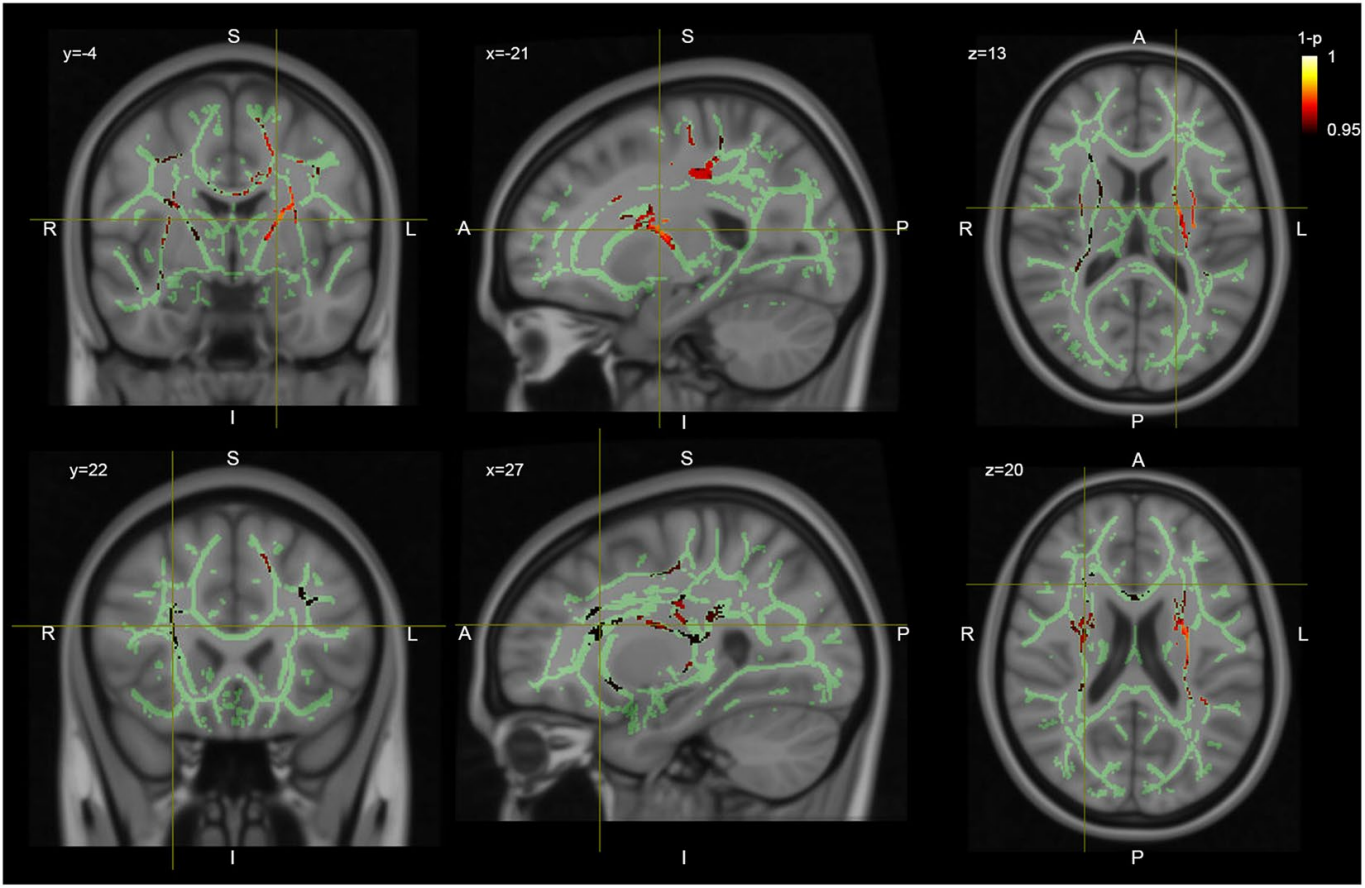
Whole brain differences in FD HC > SZ. TBSS results of the comparison of the FD values between SZ patients and healthy controls. Clusters exhibiting statistically significant decreases in the patient population (p < 0.05) are shown in red on the green TBSS FA skeleton.

Patients with chronic SZ showed a significantly reduced FD in several brain regions compared to HC (Fig. 2 and Table 2). The affected tracts were the right and left TR, the right and left SLF, the right and left corticospinal tract (CST), the body of the corpus callosum (CC), and the right inferior fronto-occipital fasciculus (IFOF). In contrast, no significant group differences in FA were detectable at a whole-brain level. However, with a more liberal threshold of p < 0.1, FA difference between chronic SZ patients and HC were detectable in the similar brain regions of our FD analysis. No increased FD or FA values were found in patients with chronic SZ compared to the HC group.

**Table 2:** Significant TBSS whole brain FD group differences HC > SZ.

Cluster Size (Voxel)	Max X	Max Y	Max Z	Structures to which each cluster belongs to*
5678	−21	−5	14	Superior longitudinal fasciculus, Corticospinal tract, Thalamic radiation
2914	31	−15	24	Superior longitudinal fasciculus, Inferior fronto-occipital fasciculus, Corticospinal tract, Inferior longitudinal fasciculus, Thalamic radiation, Uncinate fasciculus
199	27	22	20	Inferior fronto-occipital fasciculus, Thalamic radiation

*Only tracts with >1% probability are included in the labels (tracts with highest probability are listed first) and only clusters containing >10 voxels are reported. The statistical threshold was set to whole-brain cluster-level FWE p < 0.05.

### Differences in FD and FA value between patients with first episode psychosis and patients with schizophrenia

In an explorative analysis, we tested whether the FD TBSS analysis approach enables us to detect microstructural white matter differences between chronic and early stage of the schizophrenia spectrum. We hypothesized that patients with chronic schizophrenia would show greater FD alterations compared to first episode patients. To test this hypothesis, we analyzed data from 14 patients with first episode psychosis (FEP) (for demographics see Table S2). Please note, that these data were collected with the completely same acquisition parameters as the HC and chronic SZ group. We performed a whole brain FD group comparison between patients with FEP and patients with chronic SZ. In several brain regions the FD was significantly reduced in patients with chronic SZ compared to patients with FEP (Table S3 and Figure S1). The pattern of the most affected regions were similar to our previous analysis between HC and chronic SZ patients including the left ATR; left SLF, the left CST. Again, we observed no significant group differences in FA values. Within the psychosis spectrum, our FD TBSS analysis approach revealed significant microstructural changes between patients with chronic SZ and early stages of psychosis. No significant differences were detectable between the HC and FEP patients. Please see the supplementary material section for more details regarding this analysis.

### Relation between reduced fiber density and positive psychotic symptoms

**Figure 3:**
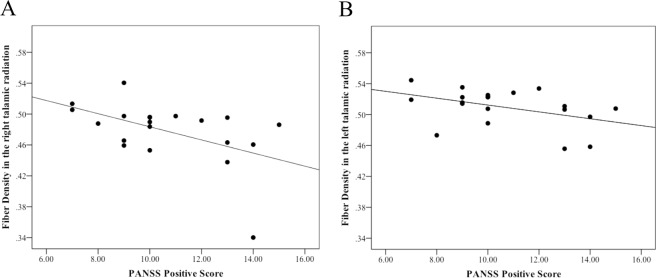
Correlation between fiber density in the thalamic radiation and positive symptoms. Spearman rank correlations (r_s_) between FD values in in the right and left thalamic radiation and positive symptoms (PANSS Positive Score) (r_s_ = −0.522, p = 0.018; left: r_s_ = −0.424, p = 0.063).

We found a significant correlation between mean FD values in the right TR and positive psychotic symptoms (r_s_ = −0.522, p = 0.018) as well as a trend-level effect in the left TR (r_s_ = −0.424, p = 0.063) in schizophrenia patients (Table 3, Fig. 3). However, when adjusting for multiple comparison, the correlation in the right TR did not survive significance (r_s_ = −0.522, Bonferroni adjusted p = 0.090). No significant correlation between positive psychotic symptoms and mean FD were observed in one of the other ROIs (Table 3). Given the fact that antipsychotic medication correlated significantly with the severity of positive symptoms (r_s_ = 0.478, p = 0.033), we performed an additional correlation analysis with chlorpromazine equivalents and mean FD values of the five predefined ROIs. Antipsychotic medication significantly correlated with mean FD values in the right TR (r_s_ = −0.516, p = 0.02). In all other regions, we did not find any significant association with antipsychotic medication (left TR, r_s_ = −0.179, p = 0.45 left SLF, r_s_ = −0.371, p = 0.107; right SLF, r_s_ = −0.364, p = 0.115).

**Table 3:** Correlations between positive psychotic symptoms and FD in predefined ROIS.

Structural ROIs	Spearman rank correlation	p-value
left TR	r_s_ = −0.424	0.063
right TR	r_s_ = −0.522	0.018
left SLF	r_s_ = −0.299	0.201
right SLF	r_s_ = −0.311	0.182
right UF	r_s_ = 0.002	0.992

Spearman rank correlations (r_s_) between FD values in predefined ROIs and positive symptoms measured with the. PANSS Positive Score.

Finally, in an explorative analysis, we investigate potential association with negative symptoms and the mean FD values in the TR and SLF. Neither the BNSS apathy factor (left TR, r_s_ = −0.131, p = 0.581; right TR, r_s_ = 0.114, p = 0.632; left SLF, r_s_ = 0.248, p = 0.291; right SLF, r_s_ = 0.226, p = 0.339; righ UF, r_s_ = −0.13, p = 0.586) nor the BNSS diminished expression factor (left TR, r_s_ = −0.223, p = 0.344; right TR, r_s_ = −0.066, p = 0.784; left SLF, r_s_ = 0.035, p = 0.885; right SLF, r_s_ = 0.083, p = 0.728, righ UF, r_s_ = −0.136, p = 0.566) correlated significantly with the FD in the TR, SLF and right UF.

## Discussion

The present study tested a novel diffusion data analysis approach to detect alterations in WM fiber density in patients suffering from SZ. We found widespread microstructural FD changes in chronic SZ patients in comparison to HCs. The reduction in FD was most prominent in frontal and subcortical regions including the TR, the SLF, CC, and CST. Critically, patients with chronic SZ showed reduced FD not only compared to HC but also in comparison to patients with early stage of psychosis. This suggests that FD alterations propagate during the disease course of schizophrenia. In contrast, FA values did not differ significantly between SZ patients and HC or SZ and patients with FEP. Thus, the FD may have advantages to identify subtle changes of WM fibers compared to the FA. This hypothesis is underpinned by the fact that reduced FA values in patients with chronic SZ observed at a more liberal threshold (p < 0.1) follow a similar regional pattern as the reduced FD findings. Finally, FD alterations in the TR were associated with the severity of positive psychotic symptoms and medication dose. The present study introduced a useful diffusion data analysis approach, which could foster the progress to identify the complex nature of WM microstructure alterations in schizophrenia. Our findings contribute to the existing MR diffusion literature suggesting that WM alterations are a structural substrate of core information-processing deficits in schizophrenia^7,8,78^.

### Benefits of FD analysis for the interpretation of microstructural correlates

Measures of structural integrity inferred from DTI-based calculations, such as the commonly used FA value, are affected by the inherent problem of the diffusion tensor. In particular, the diffusion tensor may not be able to characterize the underlying tissue structure accurately, especially in voxels containing complex fiber configurations^20–25^. The new developed FD measure benefits from recent advances of higher order diffusion models, improved tractography algorithms^35,40,44,68^, state of the art tractogram optimization techniques^46^, and microstructural diffusion models^79–81^. By combining these analyzing methods, new white matter measures such as FD can be established. Critically, these measures better represent the underlying WM fiber structure even in voxels containing complex fiber architectures and could overcome the limitations of other quantitative, tensor-derived measures (for an extensive review, see the works of Pettersson-Yeo *et al*. and Jones^3,30^). Thus, the FD might be suitable to detect subtle WM fiber alteration in schizophrenia and other neuropsychiatric disorders.

However, intra-subject variability and reproducibility are crucial issues when comparing two structural brain measures. In particular, it is important to ensure that the intra-subject variability and reproducibility of the new measure (here FD) exhibits the same characteristics as the established measure (here FA) under comparison. By comparing the CoV of the FA and the FD in an independent longitudinal data set, we provide initial evidence for a comparable accuracy of the new FD measure and the FA. These findings suggest a high reproducibility of the FD although larger non-clincial and clinical longitudinal data are needed.

### FD changes relation to frontal dysconnectivity in schizophrenia

In line with the growing evidence for altered WM integrity in frontal and temporal regions in patients with schizophrenia^1,3,9,11^ the most prominent FD reduction were observed in tracts connecting subcortical and frontal as well as temporal and frontal regions. Critically, the altered FD of the TR, ILF and IFOF is supported by meta-analytic evidence for reduced WM volume and FA in these regions^9,82^ and recent studies comparing patients with SZ and patients with bipolar disorder^83^. In addition to these reports of WM alterations, there is meta-analytic evidence for reduced frontal and thalamic grey matter volume in schizophrenia^9^. This suggests an association between altered WM microstructure in thalamo-cortical connection and reduced grey matter volume of these interconnected regions. In accordance with these consistent structural abnormalities, there is growing evidence that frontal functional dysconnectivity, in particular thalamo-cortical dysconnectivity, is a core neurobiological abnormality in schizophrenia^3,12,84^. Thalamo-cortical dysconnectivity has been consistently reported not only in chronic patients, but even in early psychosis and individuals at clinical high risk stages^85,86^. Taken together, this accumulating evidence suggests a strong association between structural and functional frontal dysconnectivity in schizophrenia. However, multimodal neuroimaging studies combining DWI with fMRI are needed to elucidate the direct interplay between structural and functional alterations.

### Frontal FD alterations as neural substrate for positive symptoms

In the present sample, deficits in WM microstructure of the fronto-thalamic pathway were most prominent in patients with higher positive symptoms. Several previous studies reported an association between positive symptoms and altered WM integrity in different fronto-temporal pathways including the TR, arcuate fasciculus, SLF, UF and IFOF^13,14,87,88^. With respect to the fronto-thalamic pathway, the association between positive psychotic symptoms and reduced FD is in accordance with a trend-wise correlation in a recent large meta-analytic DTI analysis^89^. In addition, resting state FMRI studies showed that fronto-thalamic disconnectivity is associated with psychotic symptoms^86^. Furthermore, a specific role of the arcuate fasciculus and the SLF in the development of auditory hallucinations has been proposed^14,16,17,90^. The lack of association between SLF and positive symptoms in the present study may be explained by the fact that the PANSS positive symptom score cover several items other than auditory verbal hallucinations. However, the association between positive symptoms and reduced FD in the fronto-thalamic pathway supports the hypothesis that alterations in long-range association tracts projecting in the frontal lobe may be a core structural substrate of general information processing deficits in psychosis^3,13,78^.

### Potential medication effects on FD

In the present study positive symptoms were highly associated with current antipsychotic dose and reduced FD in the TR was also correlated with antipsychotic medication. In a cross-sectional design, we are not able to differentiate between specific effects of symptom expression or potential confounding effects of higher medication doses in patients with more severe positive symptoms. A recent longitudinal study showed that reduced FA in the TR was restored after 6 weeks of amisulpride treatment (D2/3 receptor blockade)^13^ suggesting that second generation antipsychotics may protect against WM alterations^91,92^. This is supported by a recent naturalistic study in which microstructural WM changes recovered after successful treatment in drug-naïve patients^93^. However, other recent longitudinal studies are inconclusive; increase^94^, decrease^95^, or no effect^96,97^ of medication on FA values have all been reported. Furthermore, the largest DTI cross-sectional study and meta-analysis in the field to date did not find any effects of medication dosage on WM integrity^89^. However, findings from a recent small sample study suggest a medication effects depending on the different drug type (clozapine, other atypical, typical)^98^. The present study is the first investigating FD in medicated patients with SZ. Therefore, future studies in unmedicated patients and longitudinal treatment studies would be very valuable to clarify the role of antipsychotic medication on the microstructural changes in FD.

### Limitations and future directions

Some limitations of this study should be considered. First, although we observed robust whole-brain FD differences between HC and patients with chronic SZ, the small sample size might have limited our ability and power to identify specific clinical correlates of reduced FD at our given alpha level. The same issue potentially accounts for the lack of group differences between HC and patients with FEP in our explorative analysis. Future studies with larger sample sizes should try to elucidate whether FD changes are specific to frontal and temporal tracts or reflect a more global deficit in schizophrenia. Furthermore, larger studies across the complete schizophrenia spectrum are needed to investigate whether FD alterations occur only in later disease stages or are also prevalent in early psychosis and ultra-high risk states. Second, all patients received a stable dose of second-generation antipsychotics. Although, previous studies did not find any effect of medication and duration of illness on WM integrity^89^, both factors may affect the FD in patients with chronic SZ. Therefore, studies in unmedicated patients would be very valuable to identify the role of medication on FD changes. Third, in order to acquire all data within a clinically feasible scan time suitable to a clinical population, a rather short diffusion protocol with only 32 directions and a single b-value of 1000 s/mm^2^ had to be used. Several techniques were applied to overcome the limitations of the rather small number of diffusion directions: (1) a probabilistic tractography algorithm was chosen, and (2) the final tractogram for each subject was derived from 5 million streamlines, which were then optimized by the COMMIT framework. The fact that the diffusion data only consisted of a single shell did not compromise the COMMIT optimization with respect to the intracellular compartment fraction (referred to as FD). However, in order to reliably distinguish multiple isotropic compartments with different diffusion times (e.g. gray-matter and cerebrospinal fluid), it would be crucial to acquire multi-shell diffusion data. Fourth, compared to traditional, tensor-based models, the derivation of the new FD is computationally more intensive. However, all steps, except the final TBSS processing, can be executed in subject space on cloud computing infrastructures. Thus, computation time can be reduced dramatically, and state of the art computer infrastructures allow FD estimation even for large-scale data. Lastly, while there is converging evidence from structural and functional connectivity studies supporting the disconnectivity hypothesis of schizophrenia, combined DWI and fMRI studies are needed to elucidate the structural substrates of information processing deficits.

## Conclusion

The present study found that estimating FD values show great promise as detailed and quantitative measure for WM integrity changes in schizophrenia. Critically, this new FD parameter may be more suitable to detect microstructural WM changes compared to the FA value. Thus, FD detection may foster the progress to evaluate the pathological processes of altered connectivity in schizophrenia.

## Supplementary information

Supplementary Information
