# Supplementary material for: Subtle white matter alterations in schizophrenia identified with a new measure of fiber density
Source: Sci Rep. 2019 Mar 15;9:4636. doi: 10.1038/s41598-019-40070-2 (PMC6420505; doi:10.1038/s41598-019-40070-2)
Supplement: Supplementary file 1 — Supplementary Information [file 41598_2019_40070_MOESM1_ESM.pdf]

Subtle white matter alterations in schizophrenia identified with a new measure of fiber density

Philipp Stämpfli[a,b,*], Stefan Sommer[a,b*], Andrei Manoliu[a], Achim Burrer[a], André Schmidt[c], Marcus Herdener[d], Erich Seifritz[a], Stefan Kaiser[e], Matthias Kirschner[a, d]

[a]*Department of Psychiatry, Psychotherapy and Psychosomatics, Psychiatric Hospital, University of Zurich, Zurich, Switzerland*

[b]*MR-Center of the Psychiatric Hospital and the Department of Child and Adolescent Psychiatry, University of Zurich, Zurich, Switzerland*

[c]*University of Basel, Department of Psychiatry (UPK), Basel, Switzerland*

[d]*Center for Addictive Disorders, Department of Psychiatry, Psychotherapy and Psychosomatics, Psychiatric Hospital, University of Zurich, Switzerland*

[e]*Division of Adult Psychiatry, Department of Mental Health and Psychiatry, Geneva University Hospitals*

***The first and second authors have contributed equally to this work.**

Supplement

Coefficients of Variation (CoV) of FA and FD

To test whether the intrasubject variability and reproducibility of the FD measure is in the same range as the intrasubject variability of the FA measure, we assessed the coefficients of variation (CoV) of both measures in an independent longitudinal data set of 36 healthy subjects. All 36 subjects were measured on two separate time points with 7 days between the first and second scan. The acquired anatomical T1 weighted data and the diffusion data were preprocessed in the same way as described in the main material and methods section. TBSS was used to generate normalized and registered FA and FD maps for every subject and every acquisition time point. The probabilistic tractography atlas (JHU white-matter tractography atlas), which describes 20 major fiber bundles, was then applied to extract the FA and FD values for every of the 20 defined major fiber tracts, i.e., the probability tract maps served as ROIs to extract the FA and the FD values within each of the 20 fiber bundles. The mean of these values of every tract and every timepoint was then used to derive the CoV for FA and FD (see table S1). In summary, the CoV of the FD and FA were small (mean for all regions less or equal 2%). A slightly larger CoV of the FD is not surprising, as the FA is a purely tensor derived quantity. The tensor (which contains 6 degreed of freedom DOF only) is less sensitive to small signal alterations in single diffusion directions compared to the FD which is derived and based on models with much more than 6 DOF. As previously shown for the FA, these findings provide first evidence that the FD has a high intra-subject reproducibility.

Table S1. CoV values of the FA and FD in 20 major WM fiber tracts

Brain Structure / Tract	Mean intra-subject CoV of FA value (in % ± standard deviation)	Mean intra-subject CoV of FD value (in % ± standard deviation)
Left Thalamic Radiation	0.830±0.612	1.323±1.094
Right Thalamic Radiation	0.837±0.639	1.213±0.957
Left Corticospinal	0.831±0.614	1.482±1.055
Right Corticospinal	0.948±0.673	1.136±0.662
Left Cingulum Cingulate	0.889±0.644	1.309±1.200
Right Cingulum Cingulate	1.001±0.702	2.000±1.826
Left Cingulum Hippocampus	1.119±0.924	1.434±1.335
Right Cingulum Hippocampus	1.088±0.898	1.472±1.166
Callosum Forceps Major	0.894±0.851	1.141±0.908
Callosum Forceps Minor	0.968±0.752	1.576±1.117
Left IFOF	1.008±0.749	1.487±1.097
Right IFOF	0.868±0.744	1.055±0.795
Left ILF	1.060±0.890	1.557±1.038
Right ILF	1.028±0.746	1.117±0.947
Left SLF	0.900±0.632	1.570±1.325
Right SLF	1.014±0.676	1.249±1.033
Left Uncinate	1.211±1.209	1.624±1.250
Right Uncinate	0.994±1.203	1.824±1.148
Left Arcuate	0.924±0.619	1.434±1.130
Right Arcuate	1.048±0.704	1.176±0.859

Table S2. Comparison FEP patients SZ patients

	FEP patients (n = 14)	SZ patients (n = 20)	Test statistics	*p* value
Age	24.2 (5.7)	32.7 (8.3)	U = 55.5	0.003
Gender (f ,m)	3, 11	4, 16	$\chi2$ = 0.010	0.919
Handedness (r, l)	13, 1	16, 4	$\chi2$ = 1.085	0.298
Education, Years (SD)	12.4 (2.4)	12.1 (3.5)	U = 119.5	0.469
Duration of illness, Months (SD)	6.8 (7.4)	119 (87.8)	T = -4.788	<0.0001
Chlorpromazine Equivalents (mg/d)	249.9 (317.9)	493.3 (379.9)	T = -1.962	0.058
Psychopathology				
PANSS Total	43.1 (10.5)	48.9 (11.3)	T = -1.492	0.146
PANSS Positive	9.3 (1.9)	10.7 (2.4)	T = -1.8	0.081
PANSS Negative	12.2 (5.6)	15.3 (5.9)	T = -1.544	0.132
PANSS General	21.6 (4.7)	22.9 (5.3)	T = -0.714	0.48
GAF	66.1 (10.3)	55.3 (11.1)	T = 2.863	0.007
Cognition [e]				
Cognition Score	-0.15 (0.64)	-0.44 (0.94)	T = 0.975	0.338
MWT IQ	23.3 (6.0)	26.7 (6.2)	T = -1.563	0.128

Note: Data are presented as means and standard deviations. Group differences were investigated using 2-sample t tests for continuous and χ^2 tests for categorical data. For non-normally distributed data Mann–Whitney *U* tests were applied. PANSS, Positive and Negative Syndrome Scale; BNSS, Brief Negative Symptom Scale; GAF, Global Assessment of Functioning; MWT IQ, Multiple Word Test Intelligence Quotient. [a]Duration of illness included the duration of untreated psychosis and the time period since initiation of treatment. [b]BNSS Apathy = Avolition, Anhedonia, Asociality; [c]BNSS Diminished Expression = Affective Flattening or Blunting, Alogia. [d]Cognition data were z-transformed based on the data of the HC group for each test separately. The Composite cognition score was computed as the mean of the z-transformed test scores on subject level.

Table S3. Significant TBSS whole brain FD group differences patients with FEP > patients with SZ

Cluster Size (Voxel)	Max X	Max Y	Max Z	Structures to which each cluster belongs to*
7744	-15	-15	33	Forceps minor, Anterior thalamic radiation, Superior longitudinal fasciculus, Inferior fronto-occipital fasciculus, Corticospinal tract
1413	-26	-63	15	Inferior longitudinal fasciculus, Inferior fronto-occipital fasciculus, Forceps major, Superior longitudinal fasciculus
11	-32	-52	32	Superior longitudinal fasciculus

* Only tracts with >1% probability are included in the labels (tracts with highest probability are listed first) and only clusters containing >10 voxels are reported. The statistical threshold was set to whole-brain cluster-level FWE p<0.05.

Figure S1. Whole brain differences in FD patients with FEP > patients with SZ

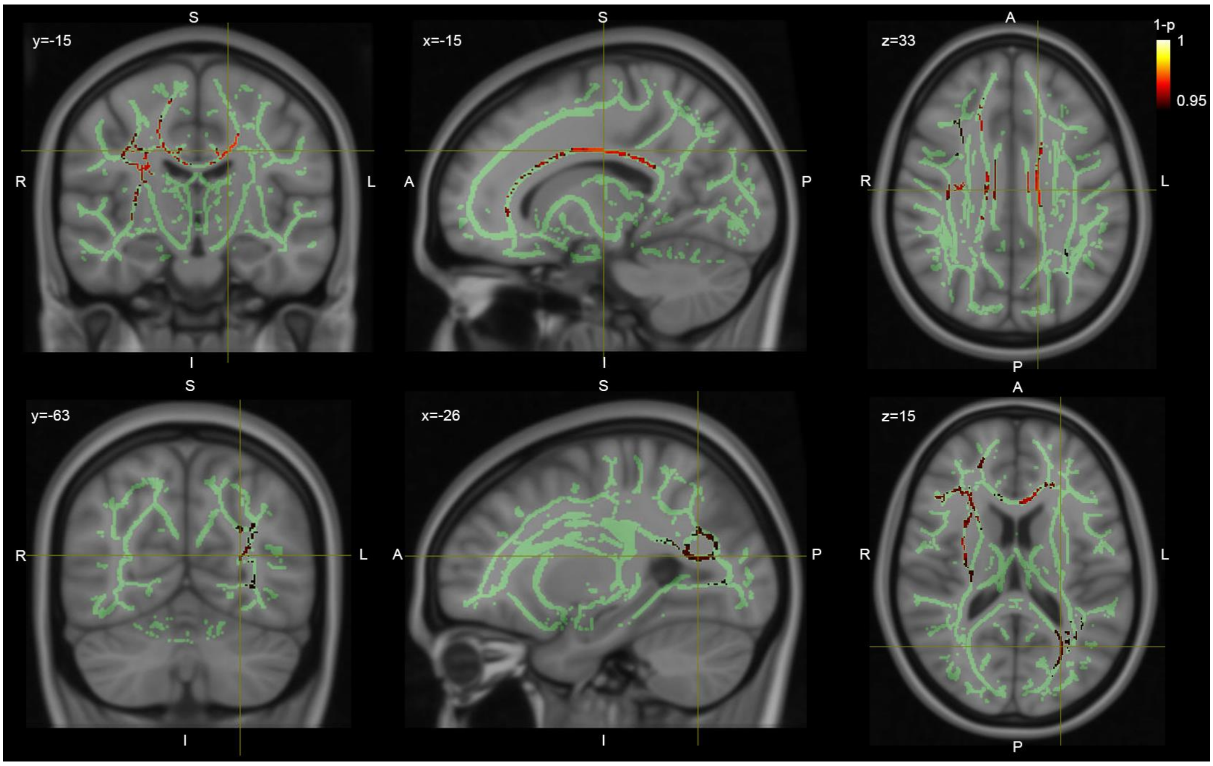

Fig S1: TBSS results of the comparison of the FD values between patients with FEP and patients with SZ. Clusters exhibiting statistically significant decreases in the SZ patient population (p<0.05) are shown in red on the green TBSS FA skeleton.
